# No damage of joint cartilage of the lower limbs in an ultra-endurance athlete – an MRI-study

**DOI:** 10.1186/1471-2474-14-343

**Published:** 2013-12-05

**Authors:** Matthias Alexander Zingg, Shila Pazahr, Fabian Morsbach, Andreas Gutzeit, Walter Wiesner, Bruno Lutz, Beat Knechtle, Thomas Rosemann, Peter Matthias Mundinger, Christoph Alexander Rüst

**Affiliations:** 1 Institute of General Practice and for Health Services Research, University of Zurich, Zurich, Switzerland; 2 University Hospital Zürich, Institute of Diagnostic and Interventional Radiology, Zurich, Switzerland; 3 Kantonsspital Winterthur, Institute of Radiology, Zurich, Switzerland; 4 Radiologie Nordost, St. Gallen, Switzerland; 5 RODIAG, St. Gallen, Switzerland; 6 Gesundheitszentrum St. Gallen, Vadianstrasse 26, St. Gallen 9001, Switzerland; 7 Hessingpark-Clinic GmbH, Institute of Radiology, Augsburg, Germany

**Keywords:** Magnetic resonance imaging, Microscopic magnetic resonance imaging, Over-use injuries, Extreme-endurance, Over-stress, Joint-injuries

## Abstract

**Background:**

Osteoarthritis is an increasing burden in an ageing population. Sports, especially when leading to an overstress of joints, is under suspicion to provoke or at least accelerate the genesis of osteoarthritis. We present the radiologic findings of a 49-years old ultra-endurance athlete with 35 years of training and competing, whose joints of the lower limbs were examined using three different types of magnetic resonance imaging, including a microscopic magnetic resonance imaging coil. To date no case report exists where an ultra-endurance athlete was examined such detailed regarding overuse-injuries of his joints.

**Case presentation:**

A 49 years old, white, male ultra-endurance athlete reporting no pain during training and racing and with no significant injuries of the lower limbs in his medical history was investigated regarding signs of chronic damage or overuse injuries of the joints of his lower limbs.

**Conclusion:**

Despite the age of nearly 50 years and a training history of over 35 years, the athlete showed no signs of chronic damage or overuse injuries in the joints of his lower limbs. This leads to the conclusion that extensive sports and training does not compulsory lead to damages of the musculoskeletal system. This is a very important finding for all endurance-athletes as well as for their physicians.

## Background

Over-stressing of joints leads to osteoarthritis. Especially in running joints of the lower limb are strained four times more than by walking [1]. The discussion whether running causes osteoarthritis led to a series of controversial results. Most authors agree that physical benefits of moderate quantity of workout exceed damages [2]. The same question for ultra-endurance running is highly contentious. Chakravarty et al. [3] reported in a prospective study that long-distance running, *i.e.* more than 300 minutes of vigorous exercise among healthy older individuals, was not associated with accelerated radiographic osteoarthritis. Since no study investigated a potential damage of joint cartilage in ultra-marathoners, we investigated whether excessive training and competing in an ultra-endurance athlete over years leads to signs of osteoarthritis using MRI (Magnetic Resonance Imaging). We determined the thickness of the articular cartilage of the lower limb for signs of osteoarthritis using conventional and microscopic MRI techniques. We hypothesized that an asymptomatic athlete training and competing for years at international level would not present alterations in joint cartilage of the lower limb.

## Case presentation

### The athlete

**Figure 1:**
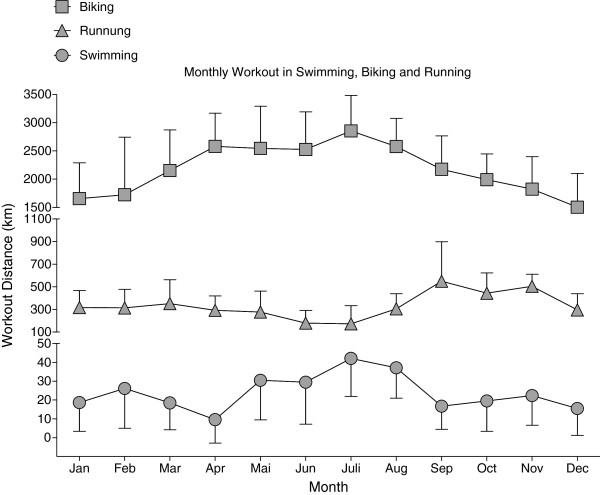
Monthly workout in swimming, cycling and running.

The 49-years old athlete started intensive swimming at the age of eight years, running by the age of 14 years and cycling at 18 years. Up to the age of 23, he competed up to national level in 50- and 100 m sprint swimming. After the age of 30 he started recording his daily exercise schedule for swimming, running and cycling. During summer time (*i.e.* May thought August), cycling and swimming distances were higher while running distance was lower than the annual average [Figure 1]. Over an 18-year period from 1995–2012 he annually averaged around 25,000 km in cycling, around 4,000 km in running and around 275 km in swimming distance [Figure 1].

**Figure 2:**
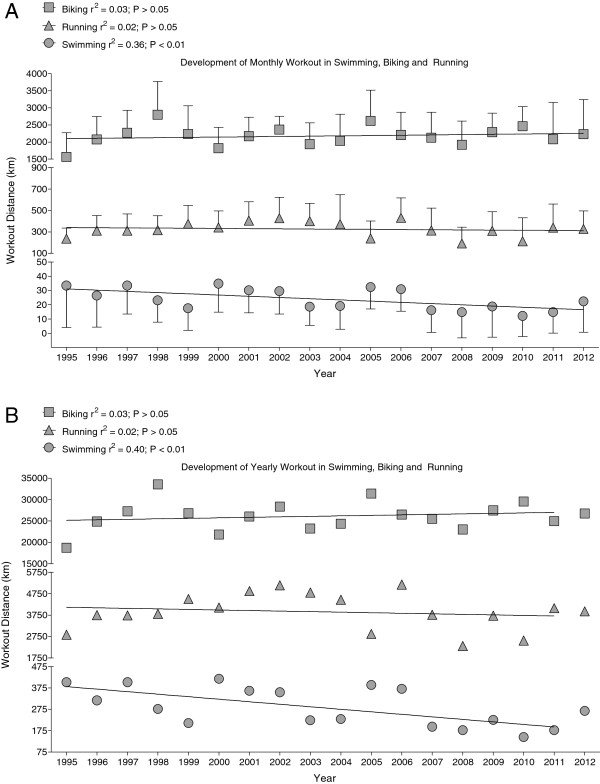
Development of monthly workout in swimming, cycling and running.

Regarding changes in exercise volumes across years, cycling and running distances were constant while his annually swimming distance decreased significantly [Figure 2]. During this 18-year period he finished 98 ultra-endurance events or 5.4 ± 3.7 events per year [Table 1]. In 2013, he was even able to win a Deca Iron ultra-triathlon (*i.e.* 10 times the Ironman distance within 10 days) within 129 h and about 6 h ahead of the second finisher.

**Table 1:** Events finished per year

**Year/race**	**Iron triathlon**^**1**^				**Ultra-running**		**Cycling**	**Swimming**		**Other**^**2**^
	**Double**	**Triple**	**Quintuple**	**Deca**	**6-h**	**12-h**	**24-h**	**26.4 km**	**12-h**	
**1995**										4
**1996**							1			3
**1997**		1					1			1
**1998**		3				1	1			
**1999**	3	2								
**2000**	4	3		1		1			1	
**2001**	2	1							1	
**2002**	4	2		1		1		1		5
**2003**	3	2			2		1	1	1	2
**2004**	1	2		1		1		1		1
**2005**	1	2	1			1	1	1	1	1
**2006**	1	2		1		1		1		1
**2007**		1	1						1	
**2008**								1		
**2009**										
**2010**		1	1					1		
**2011**	1	1				2				
**2012**		1			1	1				
**Total**	20	24	3	4	3	9	5	7	5	18

^1^Iron Triathlon = 3.8 km swimming, 180 km cycling, 42.195 km running; ^2^ other = other ultra-endurance events.

So far in his career he never suffered any significant injuries of the lower limbs and never experienced any osteoarthritis symptoms. The subject is in an excellent physical condition (1.77 m body height, 77 kg body weight, BMI 24.6 kg/m^2^, nonsmoker, no arthralgia) with no relevant family history of osteoarthritis or other diseases of the musculoskeletal system. Besides radiological examination of the articular cartilage the athlete was examined by a chiropractor. No clinical signs of articular damage were found.

## Methods

**Figure 3:**
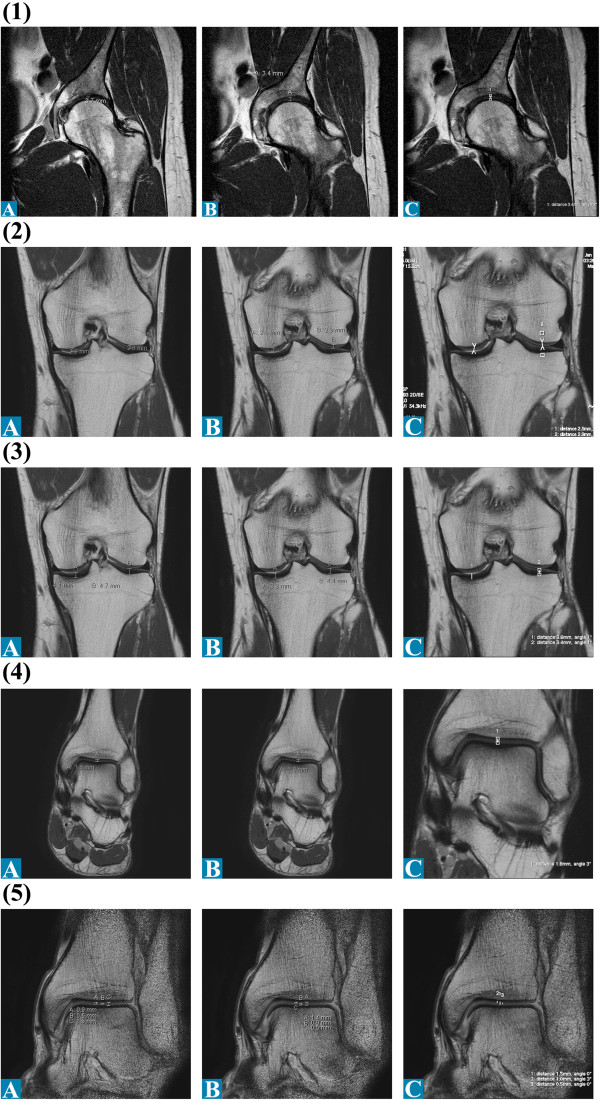
Articular cartilage thickness measurements of the left leg of radiologists A-C for 1.5 T MRI: (1) Hip coronal; (2) Femur coronal; (3) Tibia coronal; (4) and (5) UAJ coronal.

**Figure 4:**
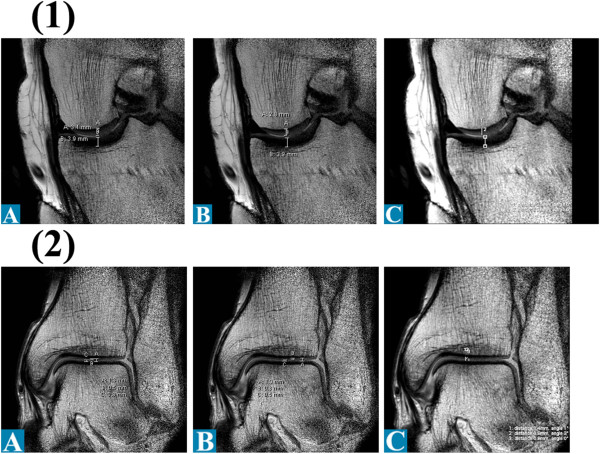
Articular cartilage thickness measurements of the left leg of radiologists A-C for Microscopy coil set: (A) Knee sagittal, (2) UAJ coronal.

**Figure 5:**
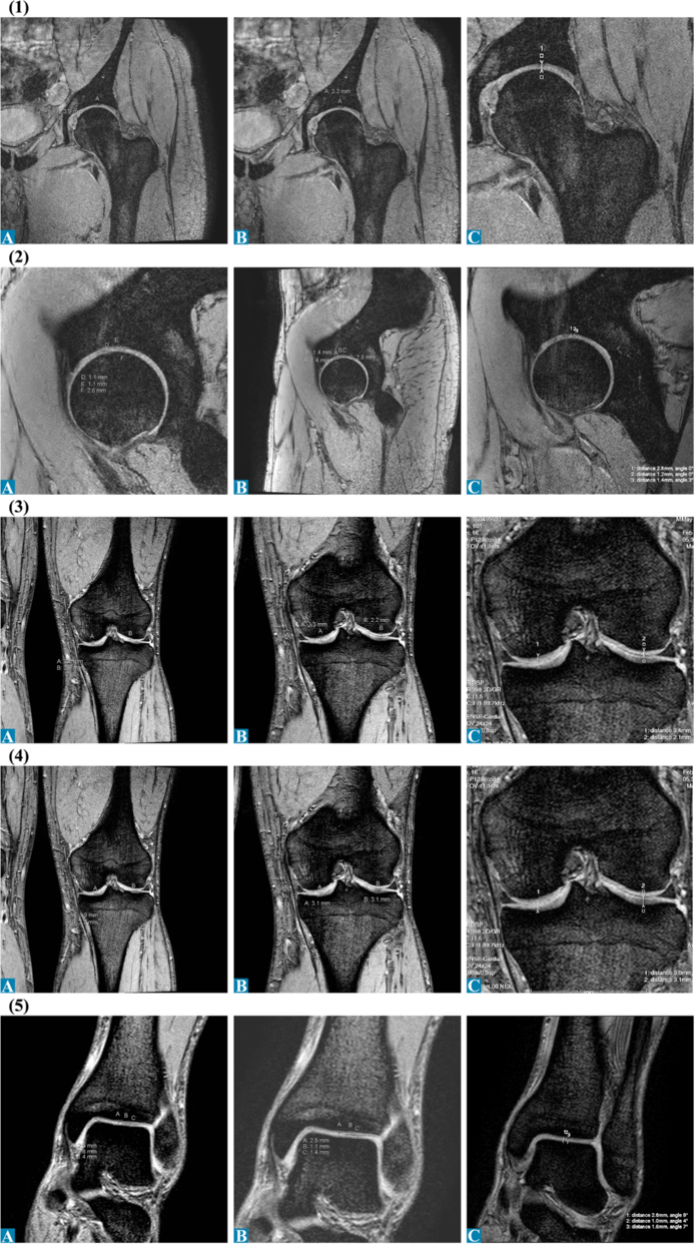
Articular cartilage thickness measurements of the left leg of radiologists A-C for 3.0 T MRI: (1) Hip coronal; (2) Hip sagittal; (3) Femur coronal; (4) Tibia coronal; (5) UAJ coronal.

**Figure 6:**
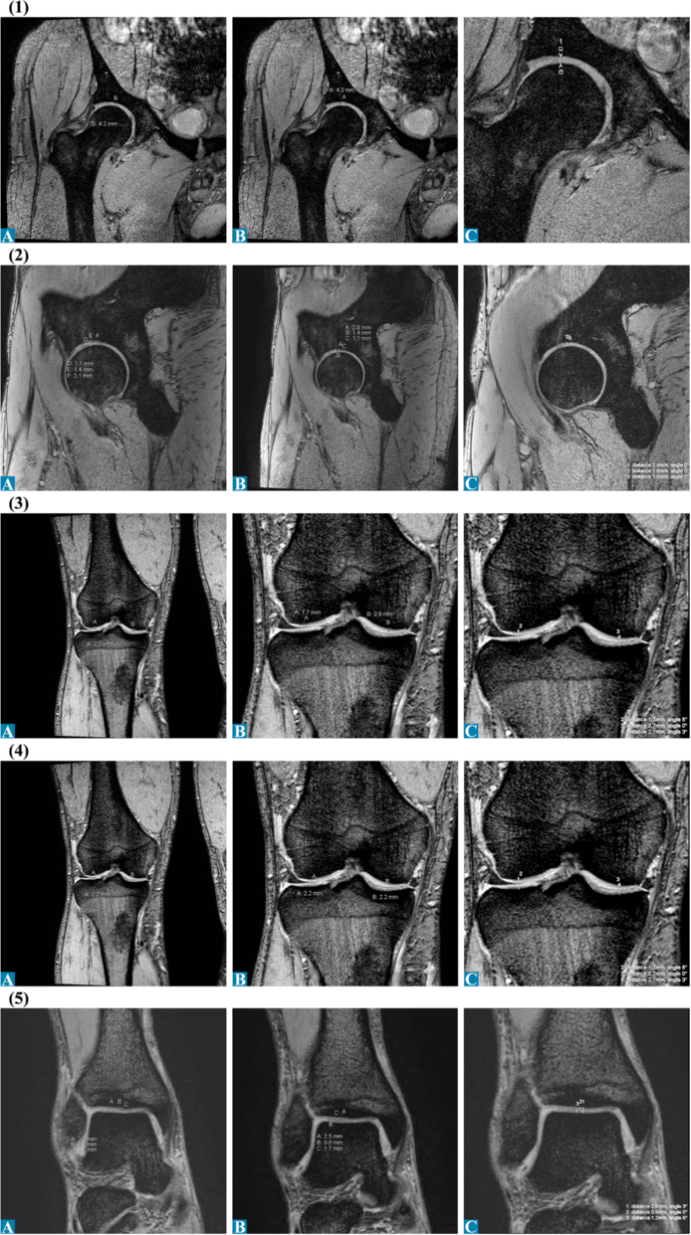
Articular cartilage thickness measurements of the right leg of radiologists A-C for 3.0 T MRI: (1) Hip coronal; (2) Hip sagittal; (3) Femur coronal; (4) Tibia coronal; (5) UAJ coronal.

The athlete underwent MR (magnetic resonance) Imaging of the three major joints of his left lower extremity. Totally, three series of MR scans were performed in two different devices with field strength of 1.5 Tesla (T) (conventional and with microscopic MRI coil) as well as 3.0 T (conventional only). The 1.5 T MR-scanner (Intera, Philips, Netherlands) provided for image acquisition of hip, knee and ankle. The conventional images were taken T2-weighted with a slice thickness of 3.0/0.3 mm. Other specifics include TE 30, TE 1929, TSE-factor 9, NSA: 6. FOV 150/2.3. Figure 3 (1,5 T MRI conventional coil set). The microscopy coil set (Intera, Philips, Netherlands) enabled small field of view imaging with high in-slice resolution. The images were taken T2-weighted with a slice thickness of 1.0/0.3 mm. Figure 4 (1,5 T MRI microscopy coil set). The 3.0 T MR-scanner (Best, Philips, Netherlands) provided for image acquisition of hip, knee and ankle. The images were taken T2-weighted with a slice thickness of 3.0/0.3 mm. Other specifics include FFE, FOV 240 mm. Figures 5 and 6 (3,0 T MRI conventional coil set, left leg and right leg). The 3.0 T Scanner were found to be accurate and tended to be more reproducible than at 1.5 T [4]. Therefore, we wanted to investigate the measured cartilage thickness in the three different techniques. The images taken were given to three different independent radiologists to measure the joint cavity, *i.e.* the cartilage thickness and to assess the grade of cartilage damage. The values for cartilage thickness are given as a mean value of the measurements of the three radiologists with the corresponding standard deviation (SD). Additionally, a fourth radiologist was asked for his opinion on the general condition of the cartilages and joints. Slight differences in cartilage thickness exist between the different modalities of MRI. Their results are listed in Table 2.

**Table 2:** Cartilage thickness of the hip, knee and upper ankle joint

		**Type and strenght (Tesla)**					
		**Standard 1,5 T**		**Standard 3,0 T**		**Micro 1,5 T**	
**Anatomy**	**Location**	**Mean (mm)**	**SD (mm)**	**Mean (mm)**	**SD (mm)**	**Mean (mm)**	**SD (mm)**
Hip cor^1^		3.5	0.2	3.5	0.3		
Hip sag^2^	Sup^4^			1.2	0.2		
Hip sag^2^	Inf^5^			1.3	0.2		
Hip sag^2^	Trans^6^			2.7	0.1		
Knee	Cond fem^7^ lat^8^	2.8	0.2	2.1	0.1		
Knee	Cond fem^7^ med^9^	2.7	0.2	3.2	0.3	2.9	0.2
Knee	Cond tib^10^ lat^8^	4.2	0.7	2.8	0.5		
Knee	Cond tib^10^ med^9^	3.9	0.2	2.7	0.7	3.9	0.0
UAJ^3^	Sup^4^	0.9	0.1	1.0	0.2	0.7	0.2
UAJ^3^	Inf^5^	0.5	0.0	1.5	0.1	0.7	0.2
UAJ^3^	Trans^6^	1.5	0.1	2.5	0.1	1.4	0.2

^1^coronal; ^2^saggital; ^3^Upper ankle joint; ^4^superior; ^5^inferior; ^6^transversal; ^7^Condylus femoralis; ^8^lateral; ^9^medial; ^10^Condylus tibialis.

## Discussion

We hypothesized that there was no significant articular cartilage damage in our athlete, analyzed by MRI. Signs for articular cartilage damage were neither found in clinical nor in radiological examinations. Three different modalities of MRI and four radiologists evaluated the images and found no articular cartilage damage to the joints.

After four decades of excessive work-out a certain level of damage to the joints, *i.e.* osteoarthritis would be expected. Osteoarthritis is characterized by changes in the structure and function of the joint [5]. Osteoarthritis develops consequently as joint cartilage softens, fibrillates and is lost [6]. Classical clinical symptoms include joint stiffness, pain and dysfunction that bring the patient generally to the physician [7]. To exclude other possible diagnosis, radiological imaging is generally performed, whereas blood tests do not play a relevant role [8]. While conventional X-ray is the most often used imaging technique to visualize osteoarthritis, MR imaging nowadays takes an important role in osteoarthritis research. The American College of Radiologists lists conventional x-rays as the gold standard for chronic hip, knee and ankle pain [9]. An ever increasing number of new and sophisticated imaging sequences and protocols in MRI provide for wholly new possibilities to quantify and define osteoarthritis [10]. For example with a dedicated coil for ‘microscopic MRI’ a higher signal yield compared to conventional MRI coils and thus a much higher signal-to-noise ratio (SNR) can be achieved, especially when performing examinations with a 1.5 T MR-scanner. This leads to the possibility of acquiring images with thinner layer thickness and higher resolution. For example it is possible to perform turbo spin echo sequences, which are commonly used to get images of cartilage tissue, with a voxel size of 1.5 mm (layer thickness) to ~0.25 mm in the square (in plane). Since a cartilage layer thickness of about 1 to 2 mm is not uncommon, even in younger athletes, the highest possible resolution (especially in plane) is mandatory to detect small defects such as cartilage delamination with formation of microscopic fluid layers in the osteochondral interface. Also for exact measuring of cartilage layer thickness and thus early detection of premonitory cartilage lesions a small voxel size leading to a high resolution is absolutely crucial.

The technical implementation of an examination with a ‘microscopic MRI coil’ is not considerably different from an examination with a conventional local coil. The coil is placed to the regions to be examined, connected to the machine and the examination can be started as usual using the respective parameters. The most impairing limitation of ‘microscopic MRI’ is the considerable low tissue penetration of about only 2.5-3 cm, but provides a significant higher signal to noise ratio (SNR). A key to solve this problem could be an arrangement of four circumferential arranged coils around the structure to be examined. The use of such an arrangement in combination with a 3 T MR-scanner and turbo spin echo sequences permits in experimental settings performed with prototypes an even higher resolution with voxel sizes up to 1.0 × 0.2 × 0.2 mm^3^.

Different radiological protocols provide for different measurements of joint cartilage [11]. Up to now no consensus exists how and at which part of the joint to measure the cartilage. Furthermore, some authors suggest measuring the thickness [12] while others concentrate on the cartilage volume [13]. To determine now whether the cartilage was damaged by excessive trainings through many years, reference values would be preferable. Up to now, reference values are not yet clinically applicable as inter-individual difference in cartilage thickness is too large [11,14]. Only a few studies addressed reference values [15] whereas most investigated the knee. An important problem provides the different modalities of MR Imaging, as slice thickness and used magnetic field strength varies with each study. Compared to published cartilage thickness for men >45 years, our athlete’s cartilage was of a similar thickness [6]. For example the cartilage of the left medial femur condylus was 2.7 ± 0.2 mm in 1.5 T, which is comparable with 3 mm from Eckstein et al. [4]. Generally the integrity of the cartilage is measured using signs of edema or fibrillation of the cartilage [16]. In the present athlete the three radiologists found none of these pre-clinic radiological signs. The fourth radiologist stated the joints to look younger than expected of a 49-year old man.

A very popular argument against running is that it causes injuries and subsequently osteoarthritis. The most common sub-acute running injuries were identified as the medial tibia stress syndrome, Achilles tendinopathy and plantar fasciitis [17]. In question of long-term injuries running is generally blamed of causing a/o accelerating osteoarthritis. ‘No sports’ from W. Churchill went down in history as the most popular anti-sport quote. Both physicians and radiologists repeatedly addressed the topic. Even often proved wrong for moderate quantities [3], common sense tells us that at least excessive running should damage lower extremity joints. Existing evidence on whether long-term long-distance running causes osteoarthritis is insufficient for researchers to draw unequivocal conclusions [18]. Chakravarty et al. [3] found in a prospective study that long-distance running among healthy older individuals was not associated with accelerated osteoarthritis. These data raise the possibility that severe osteoarthritis may not be more common among runners. Our athlete provides an extraordinary example for a healthy ultra-endurance runner.

The athlete practiced all three classical triathlon disciplines. Swimming is known to be healthy as many different muscle groups are used at the same time [19]. Furthermore, cycling is a concentric while running is an eccentric sports discipline. While running burdens the joints, cycling and swimming protects joints as much less weight most be resisted [20]. This could be an important difference between ultra-runners and ultra-triathletes as the later practice long hours of not overstraining exercise as swimming and cycling.

### Limitations

The athlete observed in this study was a 49 years old, well trained man who never suffered from prolonged pain during training and showed no significant injury of the lower limbs in his medical history. Additionally, it is obvious that anatomical conditions such as varus-valgus deformations lead to an increased strain of the involved joints and thus to an increased risk of osteoarthritis in case of additional stress caused by sports like running. Thus it must be assumed that the outcome of this study would be different if the athlete was not in such extraordinary good physical conditions.

## Conclusion

This case shows that running more than approximately 10 km and cycling 70 km per day over two decades did not lead to osteoarthritis of the joints of the lower limb. Properly trained and muscle-wise well-balanced athletes can maintain training at a high level for many years. The combination of running and cycling may prevent from articular cartilage damage in ultra-endurance athletes. We should never lose sight of the fact that running brings not just fitness but mental and physical balance too. In the 21^st^ century, this is almost invaluable to our daily lives.

## Consent

We confirm that the patient has given his written consent for the case report to be published. A copy of the written consent may be requested by the editorial office at any time.

## Competing interests

The authors declare that they have no competing interests.

## Author’s contributions

MAZ participated in the design of the study and drafted the manuscript. SP and FM interpreted the MR images as radiological experts and helped drafting the manuscript. AG and PMM provided extraordinary expertise in interpreting the images as radiological experts. WW and BL performed MR imaging and helped drafting the manuscript. BK participated in the design of the study and helped drafting the manuscript. TR and CAR provided additional expertise in general medicine as well as in sports medicine. All authors read and approved the final manuscript.

## Pre-publication history

The pre-publication history for this paper can be accessed here:

http://www.biomedcentral.com/1471-2474/14/343/prepub
